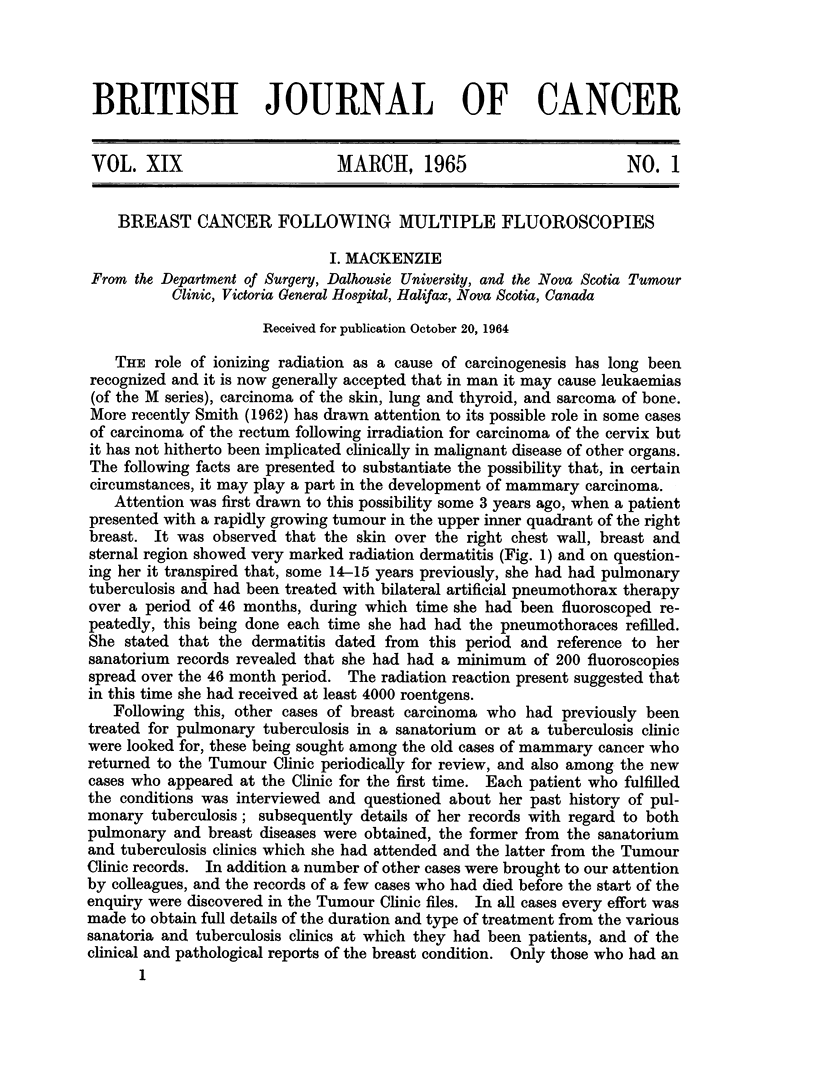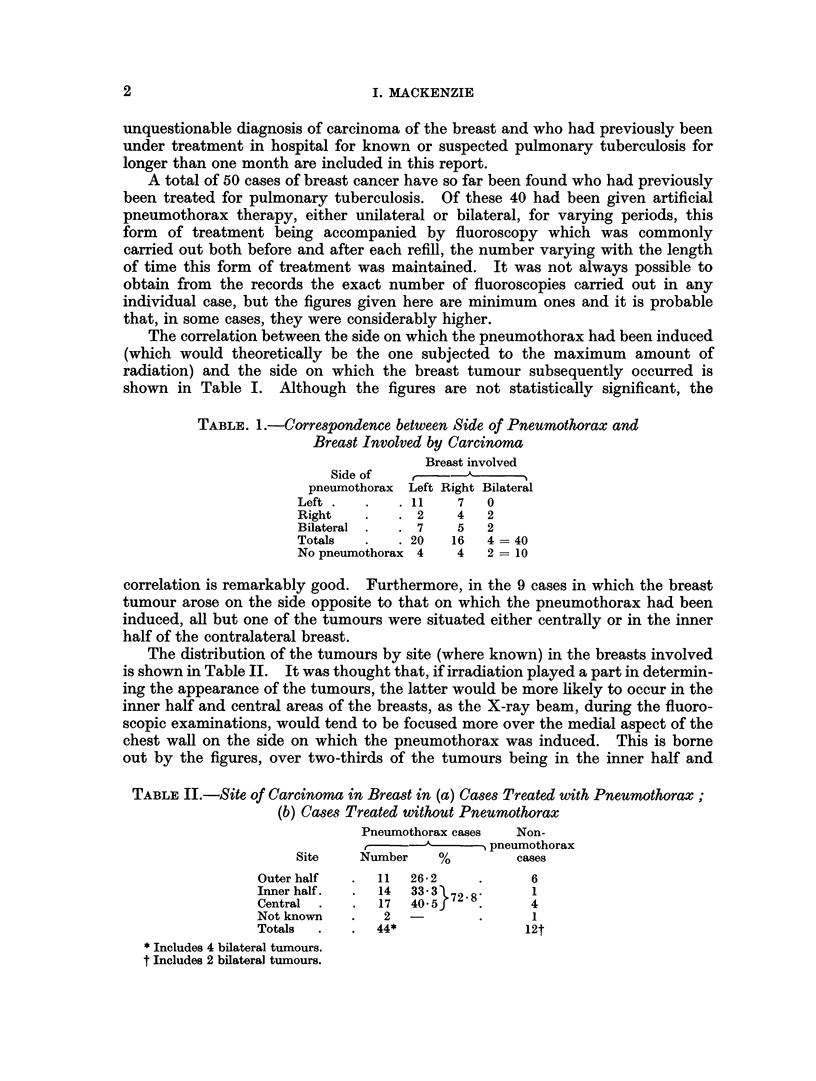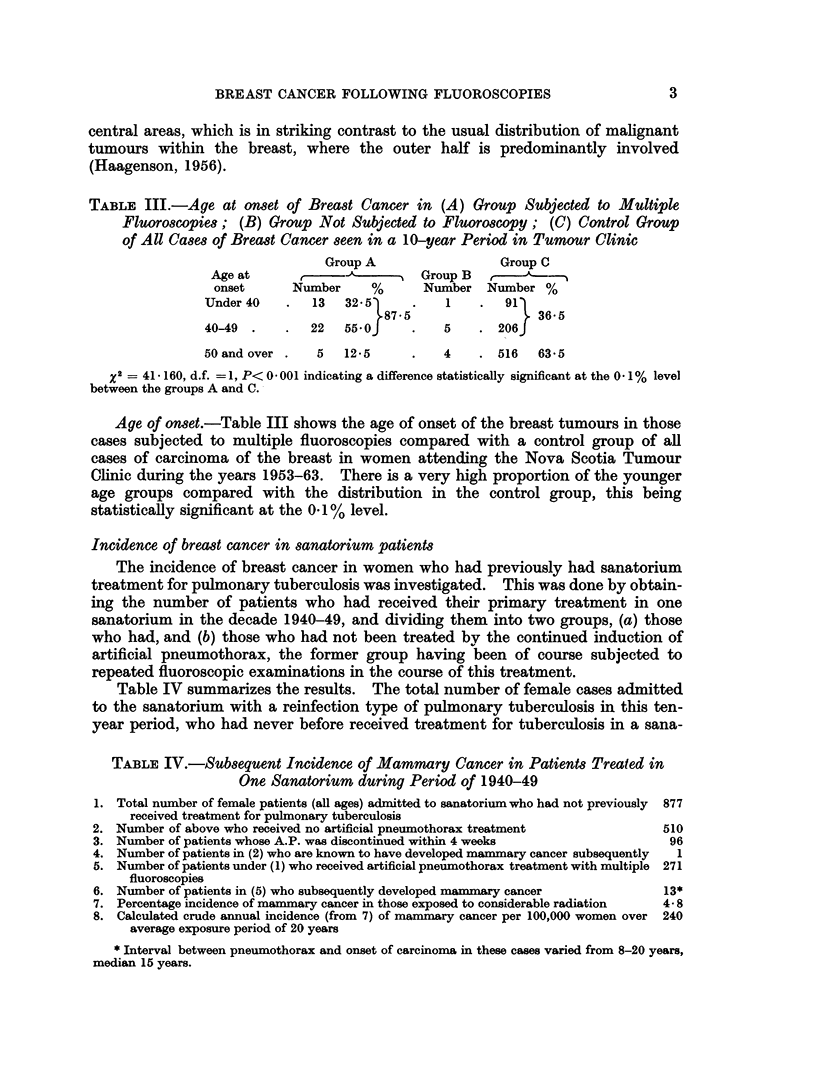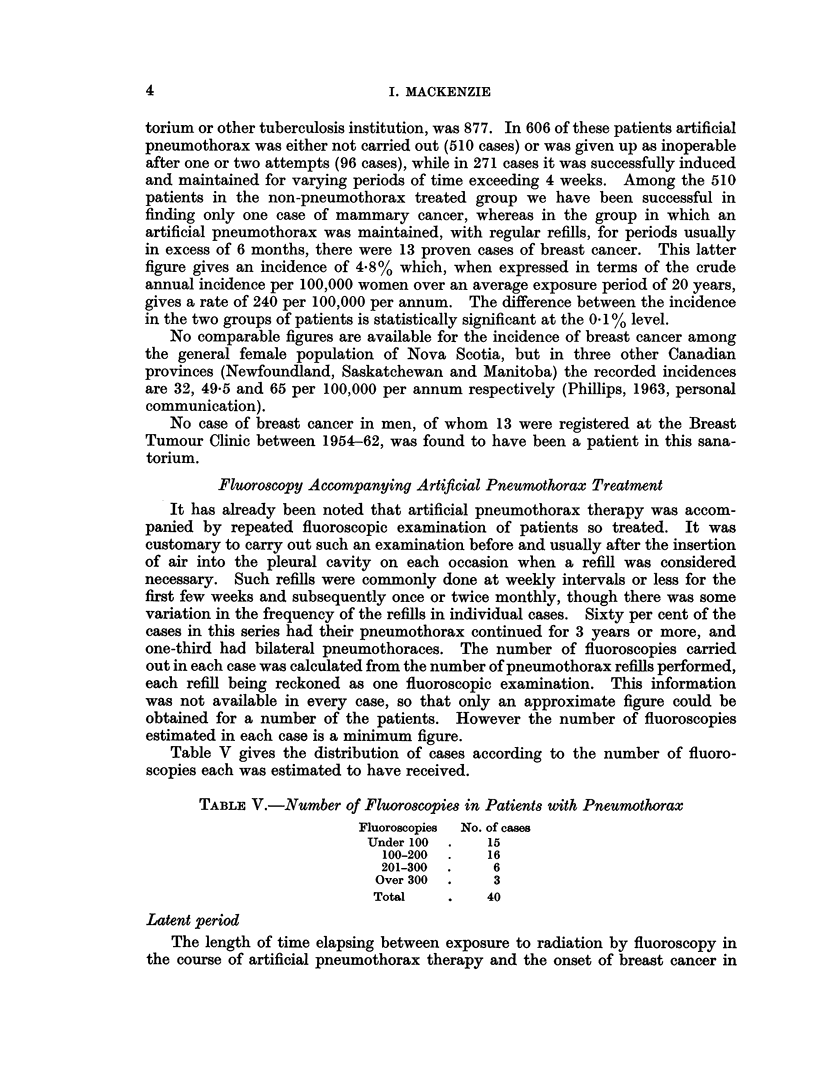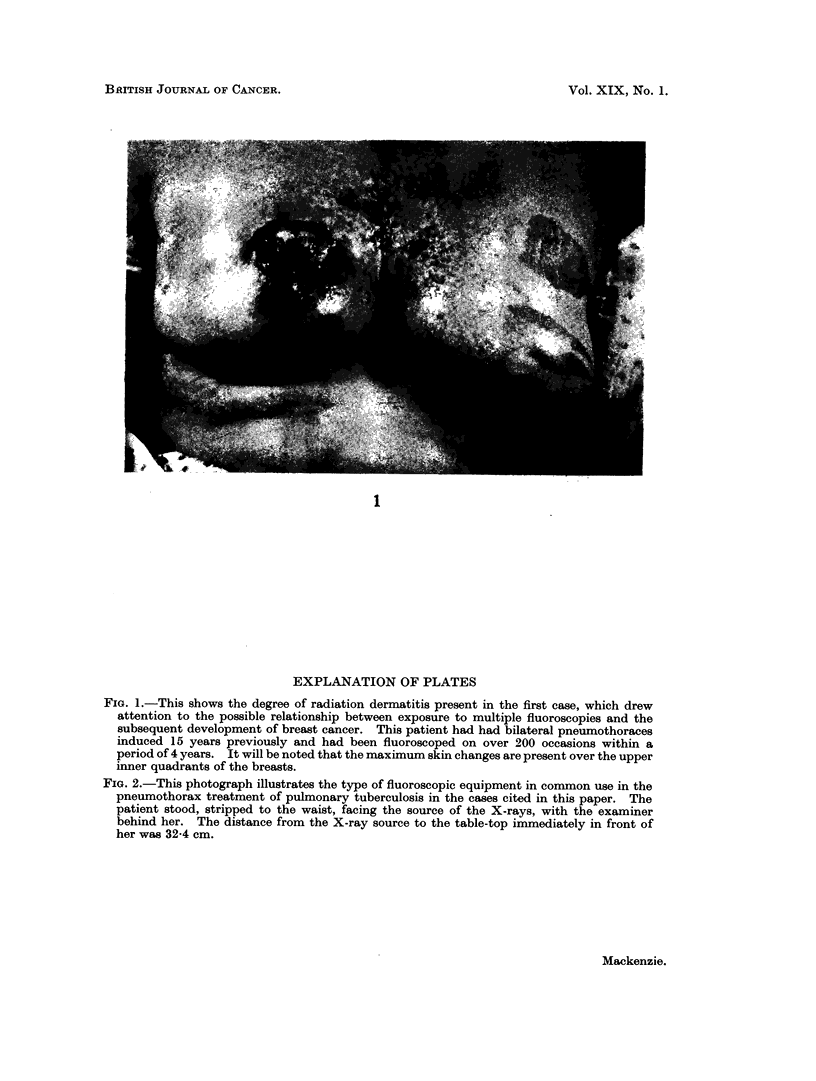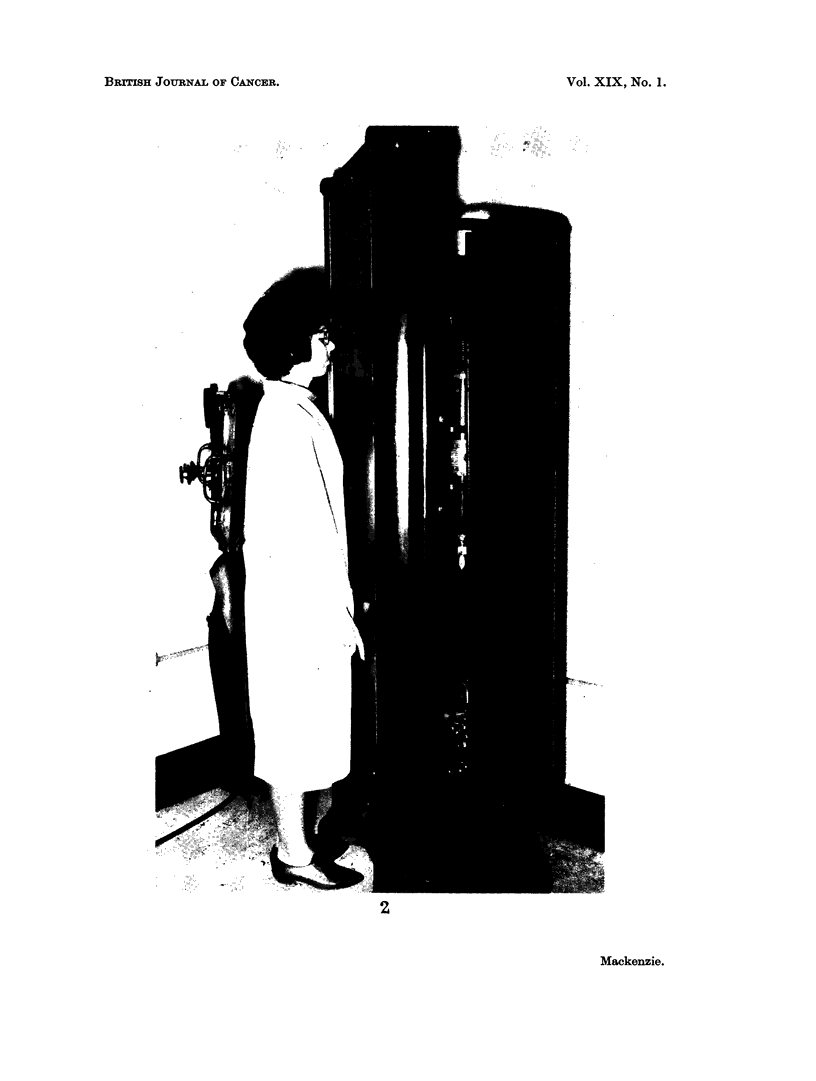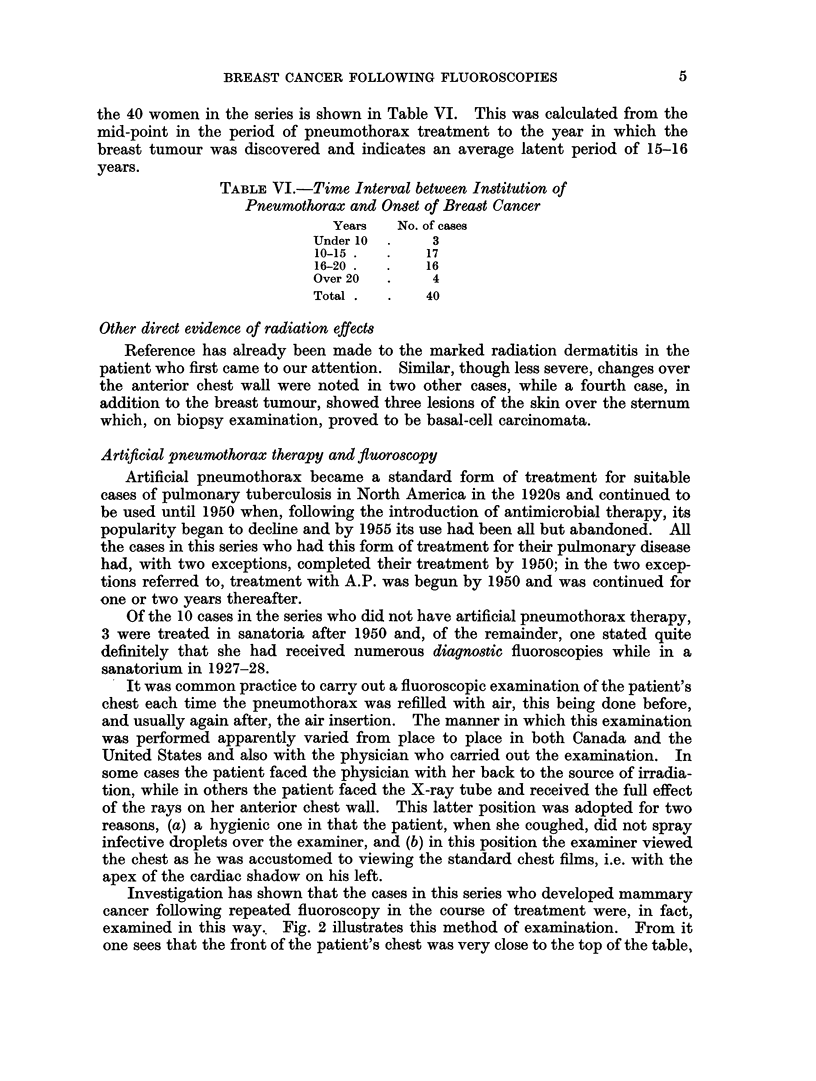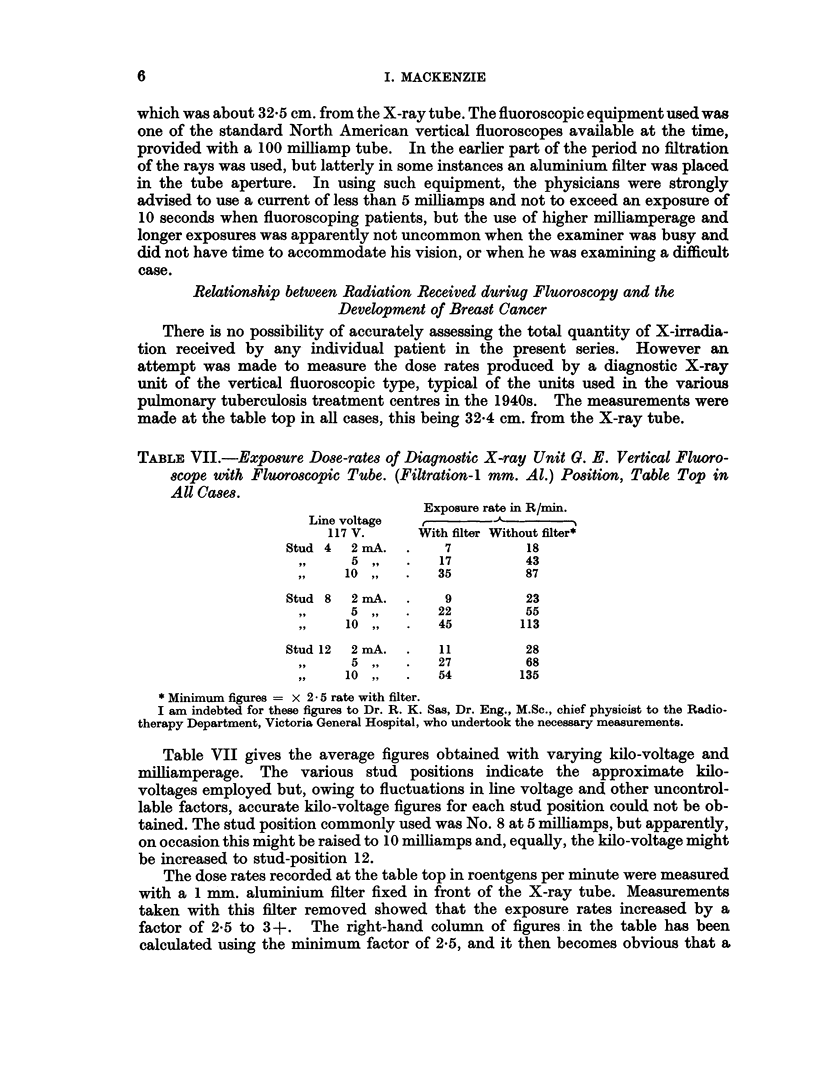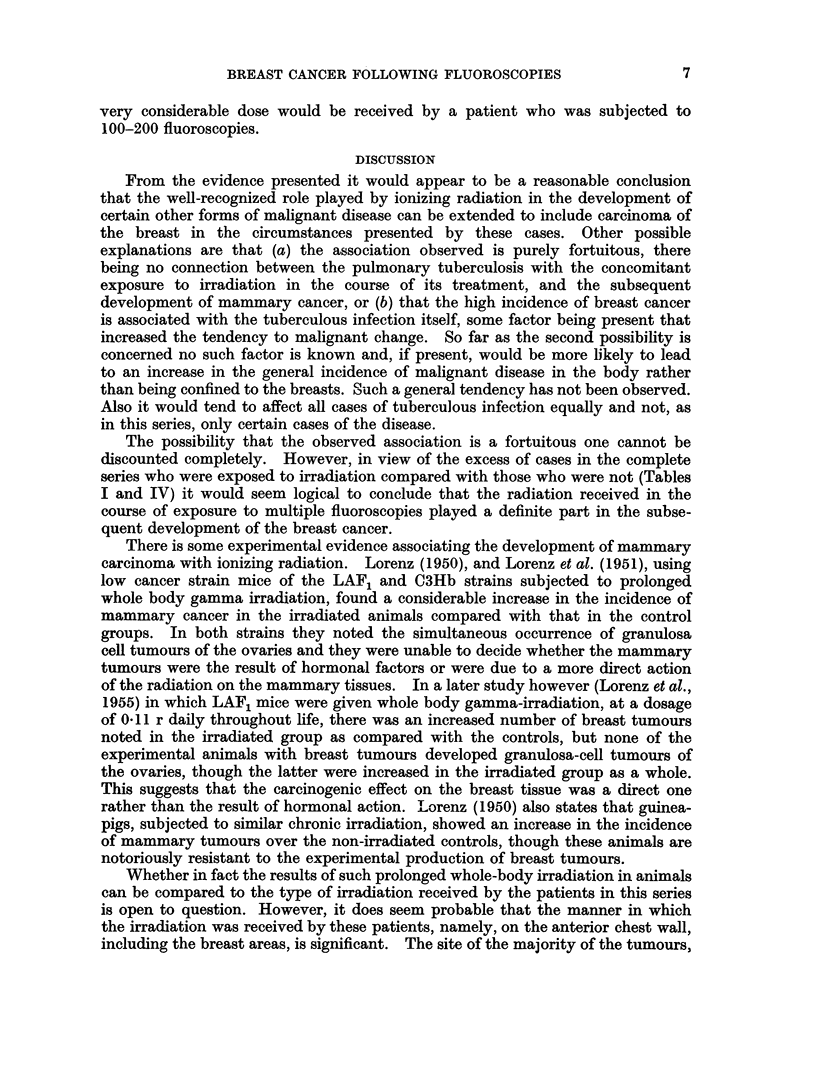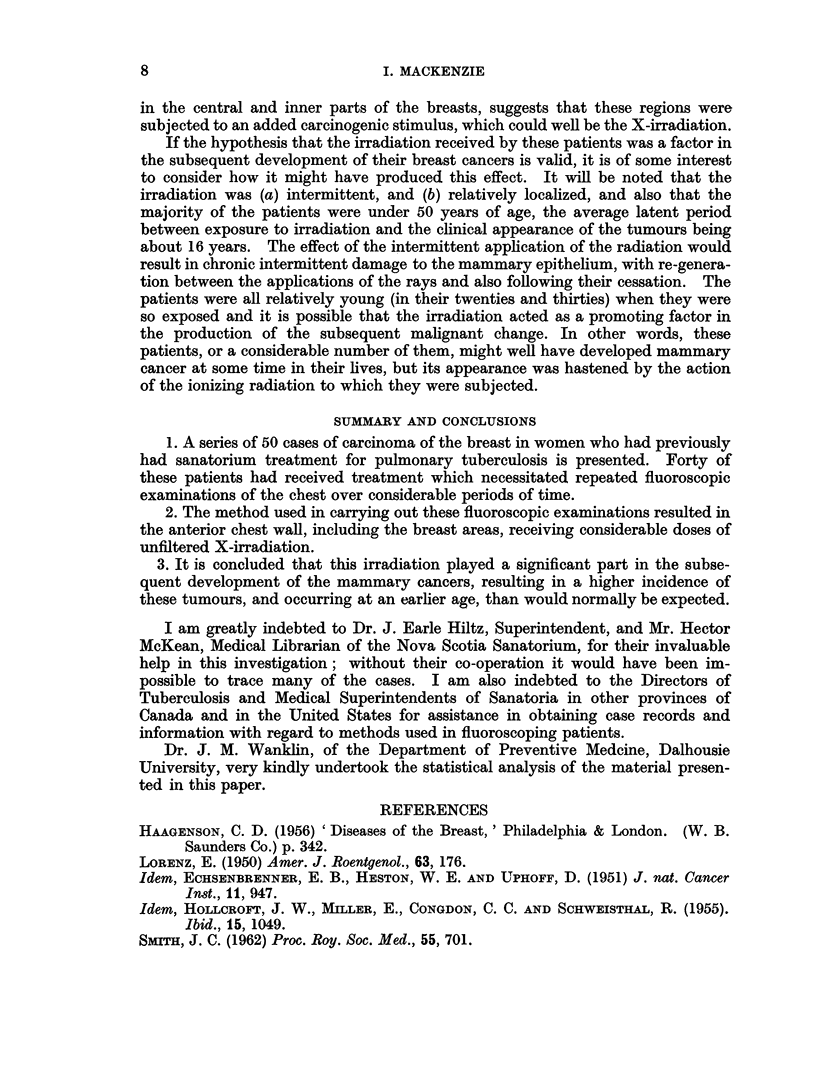# Breast Cancer Following Multiple Fluoroscopies

**DOI:** 10.1038/bjc.1965.1

**Published:** 1965-03

**Authors:** I. Mackenzie

## Abstract

**Images:**


### BRITISH JOURNAL OF CANCER

VOL. XIX MARCH, 1965 NO. 1

### BREAST CANCER FOLLOWING MULTIPLE FLUOROSCOPIES

I. MACKENZIE

*From the Department of Surgery, Dalhousie University, and the Nova Scotia Tumour Clinic, Victoria General Hospital, Halifax, Nova Scotia, Canada*

Received for publication October 20, 1964

THE role of ionizing radiation as a cause of carcinogenesis has long been recognized and it is now generally accepted that in man it may cause leukaemias (of the M series), carcinoma of the skin, lung and thyroid, and sarcoma of bone. More recently Smith (1962) has drawn attention to its possible role in some cases of carcinoma of the rectum following irradiation for carcinoma of the cervix but it has not hitherto been implicated clinically in malignant disease of other organs. The following facts are presented to substantiate the possibility that, in certain circumstances, it may play a part in the development of mammary carcinoma.

Attention was first drawn to this possibility some 3 years ago, when a patient presented with a rapidly growing tumour in the upper inner quadrant of the right breast. It was observed that the skin over the right chest wall, breast and sternal region showed very marked radiation dermatitis (Fig. 1) and on questioning her it transpired that, some 14–15 years previously, she had had pulmonary tuberculosis and had been treated with bilateral artificial pneumothorax therapy over a period of 46 months, during which time she had been fluoroscoped repeatedly, this being done each time she had had the pneumothoraces refilled. She stated that the dermatitis dated from this period and reference to her sanatorium records revealed that she had had a minimum of 200 fluoroscopies spread over the 46 month period. The radiation reaction present suggested that in this time she had received at least 4000 roentgens.

Following this, other cases of breast carcinoma who had previously been treated for pulmonary tuberculosis in a sanatorium or at a tuberculosis clinic were looked for, these being sought among the old cases of mammary cancer who returned to the Tumour Clinic periodically for review, and also among the new cases who appeared at the Clinic for the first time. Each patient who fulfilled the conditions was interviewed and questioned about her past history of pulmonary tuberculosis ; subsequently details of her records with regard to both pulmonary and breast diseases were obtained, the former from the sanatorium and tuberculosis clinics which she had attended and the latter from the Tumour Clinic records. In addition a number of other cases were brought to our attention by colleagues, and the records of a few cases who had died before the start of the enquiry were discovered in the Tumour Clinic files. In all cases every effort was made to obtain full details of the duration and type of treatment from the various sanatoria and tuberculosis clinics at which they had been patients, and of the clinical and pathological reports of the breast condition. Only those who had an unquestionable diagnosis of carcinoma of the breast and who had previously been under treatment in hospital for known or suspected pulmonary tuberculosis for longer than one month are included in this report.

A total of 50 cases of breast cancer have so far been found who had previously been treated for pulmonary tuberculosis. Of these 40 had been given artificial pneumothorax therapy, either unilateral or bilateral, for varying periods, this form of treatment being accompanied by fluoroscopy which was commonly carried out both before and after each refill, the number varying with the length of time this form of treatment was maintained. It was not always possible to obtain from the records the exact number of fluoroscopies carried out in any individual case, but the figures given here are minimum ones and it is probable that, in some cases, they were considerably higher.

The correlation between the side on which the pneumothorax had been induced (which would theoretically be the one subjected to the maximum amount of radiation) and the side on which the breast tumour subsequently occurred is shown in Table I. Although the figures are not statistically significant, the

TABLE. 1.—*Correspondence between Side of Pneumothorax and Breast Involved by Carcinoma*

| Side of pneumothorax | Breast involved: Left | Right | Bilateral |
|---|---|---|---|
| Left | 11 | 7 | 0 |
| Right | 2 | 4 | 2 |
| Bilateral | 7 | 5 | 2 |
| Totals | 20 | 16 | 4 = 40 |
| No pneumothorax | 4 | 4 | 2 = 10 |

correlation is remarkably good. Furthermore, in the 9 cases in which the breast tumour arose on the side opposite to that on which the pneumothorax had been induced, all but one of the tumours were situated either centrally or in the inner half of the contralateral breast.

The distribution of the tumours by site (where known) in the breasts involved is shown in Table II. It was thought that, if irradiation played a part in determining the appearance of the tumours, the latter would be more likely to occur in the inner half and central areas of the breasts, as the X-ray beam, during the fluoroscopic examinations, would tend to be focused more over the medial aspect of the chest wall on the side on which the pneumothorax was induced. This is borne out by the figures, over two-thirds of the tumours being in the inner half and

TABLE II.—*Site of Carcinoma in Breast in* (*a*) *Cases Treated with Pneumothorax ;* (*b*) *Cases Treated without Pneumothorax*

| Site | Pneumothorax cases: Number | % | | Non-pneumothorax cases |
|---|---|---|---|---|
| Outer half | 11 | 26·2 | | 6 |
| Inner half | 14 | 33·3 | 72·8 | 1 |
| Central | 17 | 40·5 | | 4 |
| Not known | 2 | — | | 1 |
| Totals | 44* | | | 12† |

* Includes 4 bilateral tumours.
† Includes 2 bilateral tumours.

central areas, which is in striking contrast to the usual distribution of malignant tumours within the breast, where the outer half is predominantly involved (Haagenson, 1956).

TABLE III.—*Age at onset of Breast Cancer in (A) Group Subjected to Multiple Fluoroscopies ; (B) Group Not Subjected to Fluoroscopy ; (C) Control Group of All Cases of Breast Cancer seen in a* 10*–year Period in Tumour Clinic*

| Age at onset | Group A Number | Group A % | | Group B Number | Group C Number | Group C % |
|---|---|---|---|---|---|---|
| Under 40 | 13 | 32·5 | 87·5 (Under 40 and 40–49) | 1 | 91 | 36·5 (Under 40 and 40–49) |
| 40–49 | 22 | 55·0 | | 5 | 206 | |
| 50 and over | 5 | 12·5 | | 4 | 516 | 63·5 |

$\chi^2 = 41 \cdot 160$, d.f. $=1$, $P < 0 \cdot 001$ indicating a difference statistically significant at the 0·1% level between the groups A and C.

*Age of onset.*—Table III shows the age of onset of the breast tumours in those cases subjected to multiple fluoroscopies compared with a control group of all cases of carcinoma of the breast in women attending the Nova Scotia Tumour Clinic during the years 1953–63. There is a very high proportion of the younger age groups compared with the distribution in the control group, this being statistically significant at the 0·1% level.

*Incidence of breast cancer in sanatorium patients*

The incidence of breast cancer in women who had previously had sanatorium treatment for pulmonary tuberculosis was investigated. This was done by obtaining the number of patients who had received their primary treatment in one sanatorium in the decade 1940–49, and dividing them into two groups, (*a*) those who had, and (*b*) those who had not been treated by the continued induction of artificial pneumothorax, the former group having been of course subjected to repeated fluoroscopic examinations in the course of this treatment.

Table IV summarizes the results. The total number of female cases admitted to the sanatorium with a reinfection type of pulmonary tuberculosis in this ten-year period, who had never before received treatment for tuberculosis in a sana-

TABLE IV.—*Subsequent Incidence of Mammary Cancer in Patients Treated in One Sanatorium during Period of* 1940–49

| | | |
|---|---|---|
| 1. | Total number of female patients (all ages) admitted to sanatorium who had not previously received treatment for pulmonary tuberculosis | 877 |
| 2. | Number of above who received no artificial pneumothorax treatment | 510 |
| 3. | Number of patients whose A.P. was discontinued within 4 weeks | 96 |
| 4. | Number of patients in (2) who are known to have developed mammary cancer subsequently | 1 |
| 5. | Number of patients under (1) who received artificial pneumothorax treatment with multiple fluoroscopies | 271 |
| 6. | Number of patients in (5) who subsequently developed mammary cancer | 13* |
| 7. | Percentage incidence of mammary cancer in those exposed to considerable radiation | 4·8 |
| 8. | Calculated crude annual incidence (from 7) of mammary cancer per 100,000 women over average exposure period of 20 years | 240 |

* Interval between pneumothorax and onset of carcinoma in these cases varied from 8–20 years, median 15 years.

torium or other tuberculosis institution, was 877. In 606 of these patients artificial pneumothorax was either not carried out (510 cases) or was given up as inoperable after one or two attempts (96 cases), while in 271 cases it was successfully induced and maintained for varying periods of time exceeding 4 weeks. Among the 510 patients in the non-pneumothorax treated group we have been successful in finding only one case of mammary cancer, whereas in the group in which an artificial pneumothorax was maintained, with regular refills, for periods usually in excess of 6 months, there were 13 proven cases of breast cancer. This latter figure gives an incidence of 4·8% which, when expressed in terms of the crude annual incidence per 100,000 women over an average exposure period of 20 years, gives a rate of 240 per 100,000 per annum. The difference between the incidence in the two groups of patients is statistically significant at the 0·1% level.

No comparable figures are available for the incidence of breast cancer among the general female population of Nova Scotia, but in three other Canadian provinces (Newfoundland, Saskatchewan and Manitoba) the recorded incidences are 32, 49·5 and 65 per 100,000 per annum respectively (Phillips, 1963, personal communication).

No case of breast cancer in men, of whom 13 were registered at the Breast Tumour Clinic between 1954–62, was found to have been a patient in this sanatorium.

### *Fluoroscopy Accompanying Artificial Pneumothorax Treatment*

It has already been noted that artificial pneumothorax therapy was accompanied by repeated fluoroscopic examination of patients so treated. It was customary to carry out such an examination before and usually after the insertion of air into the pleural cavity on each occasion when a refill was considered necessary. Such refills were commonly done at weekly intervals or less for the first few weeks and subsequently once or twice monthly, though there was some variation in the frequency of the refills in individual cases. Sixty per cent of the cases in this series had their pneumothorax continued for 3 years or more, and one-third had bilateral pneumothoraces. The number of fluoroscopies carried out in each case was calculated from the number of pneumothorax refills performed, each refill being reckoned as one fluoroscopic examination. This information was not available in every case, so that only an approximate figure could be obtained for a number of the patients. However the number of fluoroscopies estimated in each case is a minimum figure.

Table V gives the distribution of cases according to the number of fluoroscopies each was estimated to have received.

TABLE V.—*Number of Fluoroscopies in Patients with Pneumothorax*

| Fluoroscopies | No. of cases |
|---|---|
| Under 100 | 15 |
| 100–200 | 16 |
| 201–300 | 6 |
| Over 300 | 3 |
| Total | 40 |

### *Latent period*

The length of time elapsing between exposure to radiation by fluoroscopy in the course of artificial pneumothorax therapy and the onset of breast cancer in

1

### EXPLANATION OF PLATES

FIG. 1.—This shows the degree of radiation dermatitis present in the first case, which drew attention to the possible relationship between exposure to multiple fluoroscopies and the subsequent development of breast cancer. This patient had had bilateral pneumothoraces induced 15 years previously and had been fluoroscoped on over 200 occasions within a period of 4 years. It will be noted that the maximum skin changes are present over the upper inner quadrants of the breasts.

FIG. 2.—This photograph illustrates the type of fluoroscopic equipment in common use in the pneumothorax treatment of pulmonary tuberculosis in the cases cited in this paper. The patient stood, stripped to the waist, facing the source of the X-rays, with the examiner behind her. The distance from the X-ray source to the table-top immediately in front of her was 32·4 cm.

2

Mackenzie.

the 40 women in the series is shown in Table VI. This was calculated from the mid-point in the period of pneumothorax treatment to the year in which the breast tumour was discovered and indicates an average latent period of 15–16 years.

TABLE VI.—*Time Interval between Institution of Pneumothorax and Onset of Breast Cancer*

| Years | No. of cases |
|---|---|
| Under 10 . | 3 |
| 10–15 . . | 17 |
| 16–20 . . | 16 |
| Over 20 . | 4 |
| Total . . | 40 |

*Other direct evidence of radiation effects*

Reference has already been made to the marked radiation dermatitis in the patient who first came to our attention. Similar, though less severe, changes over the anterior chest wall were noted in two other cases, while a fourth case, in addition to the breast tumour, showed three lesions of the skin over the sternum which, on biopsy examination, proved to be basal-cell carcinomata.

*Artificial pneumothorax therapy and fluoroscopy*

Artificial pneumothorax became a standard form of treatment for suitable cases of pulmonary tuberculosis in North America in the 1920s and continued to be used until 1950 when, following the introduction of antimicrobial therapy, its popularity began to decline and by 1955 its use had been all but abandoned. All the cases in this series who had this form of treatment for their pulmonary disease had, with two exceptions, completed their treatment by 1950; in the two exceptions referred to, treatment with A.P. was begun by 1950 and was continued for one or two years thereafter.

Of the 10 cases in the series who did not have artificial pneumothorax therapy, 3 were treated in sanatoria after 1950 and, of the remainder, one stated quite definitely that she had received numerous *diagnostic* fluoroscopies while in a sanatorium in 1927–28.

It was common practice to carry out a fluoroscopic examination of the patient's chest each time the pneumothorax was refilled with air, this being done before, and usually again after, the air insertion. The manner in which this examination was performed apparently varied from place to place in both Canada and the United States and also with the physician who carried out the examination. In some cases the patient faced the physician with her back to the source of irradiation, while in others the patient faced the X-ray tube and received the full effect of the rays on her anterior chest wall. This latter position was adopted for two reasons, (*a*) a hygienic one in that the patient, when she coughed, did not spray infective droplets over the examiner, and (*b*) in this position the examiner viewed the chest as he was accustomed to viewing the standard chest films, i.e. with the apex of the cardiac shadow on his left.

Investigation has shown that the cases in this series who developed mammary cancer following repeated fluoroscopy in the course of treatment were, in fact, examined in this way. Fig. 2 illustrates this method of examination. From it one sees that the front of the patient's chest was very close to the top of the table, which was about 32·5 cm. from the X-ray tube. The fluoroscopic equipment used was one of the standard North American vertical fluoroscopes available at the time, provided with a 100 milliamp tube. In the earlier part of the period no filtration of the rays was used, but latterly in some instances an aluminium filter was placed in the tube aperture. In using such equipment, the physicians were strongly advised to use a current of less than 5 milliamps and not to exceed an exposure of 10 seconds when fluoroscoping patients, but the use of higher milliamperage and longer exposures was apparently not uncommon when the examiner was busy and did not have time to accommodate his vision, or when he was examining a difficult case.

*Relationship between Radiation Received duriug Fluoroscopy and the Development of Breast Cancer*

There is no possibility of accurately assessing the total quantity of X-irradiation received by any individual patient in the present series. However an attempt was made to measure the dose rates produced by a diagnostic X-ray unit of the vertical fluoroscopic type, typical of the units used in the various pulmonary tuberculosis treatment centres in the 1940s. The measurements were made at the table top in all cases, this being 32·4 cm. from the X-ray tube.

TABLE VII.—*Exposure Dose-rates of Diagnostic X-ray Unit G. E. Vertical Fluoroscope with Fluoroscopic Tube. (Filtration-*1 *mm. Al.) Position, Table Top in All Cases.*

| Line voltage 117 V. | | Exposure rate in R/min. | |
|---|---|---|---|
| | | With filter | Without filter* |
| Stud 4 | 2 mA. | 7 | 18 |
| ,, | 5 ,, | 17 | 43 |
| ,, | 10 ,, | 35 | 87 |
| Stud 8 | 2 mA. | 9 | 23 |
| ,, | 5 ,, | 22 | 55 |
| ,, | 10 ,, | 45 | 113 |
| Stud 12 | 2 mA. | 11 | 28 |
| ,, | 5 ,, | 27 | 68 |
| ,, | 10 ,, | 54 | 135 |

* Minimum figures = $\times$ 2·5 rate with filter.

I am indebted for these figures to Dr. R. K. Sas, Dr. Eng., M.Sc., chief physicist to the Radiotherapy Department, Victoria General Hospital, who undertook the necessary measurements.

Table VII gives the average figures obtained with varying kilo-voltage and milliamperage. The various stud positions indicate the approximate kilovoltages employed but, owing to fluctuations in line voltage and other uncontrollable factors, accurate kilo-voltage figures for each stud position could not be obtained. The stud position commonly used was No. 8 at 5 milliamps, but apparently, on occasion this might be raised to 10 milliamps and, equally, the kilo-voltage might be increased to stud-position 12.

The dose rates recorded at the table top in roentgens per minute were measured with a 1 mm. aluminium filter fixed in front of the X-ray tube. Measurements taken with this filter removed showed that the exposure rates increased by a factor of 2·5 to 3+. The right-hand column of figures in the table has been calculated using the minimum factor of 2·5, and it then becomes obvious that a very considerable dose would be received by a patient who was subjected to 100–200 fluoroscopies.

### DISCUSSION

From the evidence presented it would appear to be a reasonable conclusion that the well-recognized role played by ionizing radiation in the development of certain other forms of malignant disease can be extended to include carcinoma of the breast in the circumstances presented by these cases. Other possible explanations are that (*a*) the association observed is purely fortuitous, there being no connection between the pulmonary tuberculosis with the concomitant exposure to irradiation in the course of its treatment, and the subsequent development of mammary cancer, or (*b*) that the high incidence of breast cancer is associated with the tuberculous infection itself, some factor being present that increased the tendency to malignant change. So far as the second possibility is concerned no such factor is known and, if present, would be more likely to lead to an increase in the general incidence of malignant disease in the body rather than being confined to the breasts. Such a general tendency has not been observed. Also it would tend to affect all cases of tuberculous infection equally and not, as in this series, only certain cases of the disease.

The possibility that the observed association is a fortuitous one cannot be discounted completely. However, in view of the excess of cases in the complete series who were exposed to irradiation compared with those who were not (Tables I and IV) it would seem logical to conclude that the radiation received in the course of exposure to multiple fluoroscopies played a definite part in the subsequent development of the breast cancer.

There is some experimental evidence associating the development of mammary carcinoma with ionizing radiation. Lorenz (1950), and Lorenz *et al.* (1951), using low cancer strain mice of the $LAF_1$ and C3Hb strains subjected to prolonged whole body gamma irradiation, found a considerable increase in the incidence of mammary cancer in the irradiated animals compared with that in the control groups. In both strains they noted the simultaneous occurrence of granulosa cell tumours of the ovaries and they were unable to decide whether the mammary tumours were the result of hormonal factors or were due to a more direct action of the radiation on the mammary tissues. In a later study however (Lorenz *et al.*, 1955) in which $LAF_1$ mice were given whole body gamma-irradiation, at a dosage of 0·11 r daily throughout life, there was an increased number of breast tumours noted in the irradiated group as compared with the controls, but none of the experimental animals with breast tumours developed granulosa-cell tumours of the ovaries, though the latter were increased in the irradiated group as a whole. This suggests that the carcinogenic effect on the breast tissue was a direct one rather than the result of hormonal action. Lorenz (1950) also states that guinea-pigs, subjected to similar chronic irradiation, showed an increase in the incidence of mammary tumours over the non-irradiated controls, though these animals are notoriously resistant to the experimental production of breast tumours.

Whether in fact the results of such prolonged whole-body irradiation in animals can be compared to the type of irradiation received by the patients in this series is open to question. However, it does seem probable that the manner in which the irradiation was received by these patients, namely, on the anterior chest wall, including the breast areas, is significant. The site of the majority of the tumours, in the central and inner parts of the breasts, suggests that these regions were subjected to an added carcinogenic stimulus, which could well be the X-irradiation.

If the hypothesis that the irradiation received by these patients was a factor in the subsequent development of their breast cancers is valid, it is of some interest to consider how it might have produced this effect. It will be noted that the irradiation was (*a*) intermittent, and (*b*) relatively localized, and also that the majority of the patients were under 50 years of age, the average latent period between exposure to irradiation and the clinical appearance of the tumours being about 16 years. The effect of the intermittent application of the radiation would result in chronic intermittent damage to the mammary epithelium, with re-generation between the applications of the rays and also following their cessation. The patients were all relatively young (in their twenties and thirties) when they were so exposed and it is possible that the irradiation acted as a promoting factor in the production of the subsequent malignant change. In other words, these patients, or a considerable number of them, might well have developed mammary cancer at some time in their lives, but its appearance was hastened by the action of the ionizing radiation to which they were subjected.

### SUMMARY AND CONCLUSIONS

1. A series of 50 cases of carcinoma of the breast in women who had previously had sanatorium treatment for pulmonary tuberculosis is presented. Forty of these patients had received treatment which necessitated repeated fluoroscopic examinations of the chest over considerable periods of time.

2. The method used in carrying out these fluoroscopic examinations resulted in the anterior chest wall, including the breast areas, receiving considerable doses of unfiltered X-irradiation.

3. It is concluded that this irradiation played a significant part in the subsequent development of the mammary cancers, resulting in a higher incidence of these tumours, and occurring at an earlier age, than would normally be expected.

I am greatly indebted to Dr. J. Earle Hiltz, Superintendent, and Mr. Hector McKean, Medical Librarian of the Nova Scotia Sanatorium, for their invaluable help in this investigation; without their co-operation it would have been impossible to trace many of the cases. I am also indebted to the Directors of Tuberculosis and Medical Superintendents of Sanatoria in other provinces of Canada and in the United States for assistance in obtaining case records and information with regard to methods used in fluoroscoping patients.

Dr. J. M. Wanklin, of the Department of Preventive Medcine, Dalhousie University, very kindly undertook the statistical analysis of the material presented in this paper.

### REFERENCES

HAAGENSON, C. D. (1956) 'Diseases of the Breast,' Philadelphia & London. (W. B. Saunders Co.) p. 342.

LORENZ, E. (1950) *Amer. J. Roentgenol.*, **63**, 176.

*Idem*, ECHSENBRENNER, E. B., HESTON, W. E. AND UPHOFF, D. (1951) *J. nat. Cancer Inst.*, **11**, 947.

*Idem*, HOLLCROFT, J. W., MILLER, E., CONGDON, C. C. AND SCHWEISTHAL, R. (1955). *Ibid.*, **15**, 1049.

SMITH, J. C. (1962) *Proc. Roy. Soc. Med.*, **55**, 701.